# CXCR4-directed endoradiotherapy with [^177^Lu]Pentixather added to total body irradiation for myeloablative conditioning in patients with relapsed/refractory acute myeloid leukemia

**DOI:** 10.7150/thno.101215

**Published:** 2025-01-01

**Authors:** Krischan Braitsch, Theo Lorenzini, Maike Hefter, Katrin Koch, Katharina Nickel, Jan C Peeken, Katharina S Götze, Wolfgang Weber, Anne Allmann, Calogero D'Alessandria, Julia Brosch-Lenz, Florian Bassermann, Martina Rudelius, Mareike Verbeek, Matthias Eiber, Peter Herhaus

**Affiliations:** 1 Department of Internal Medicine III, School of Medicine and Health, Technical University of Munich, Munich, Germany.; 2 Department of Nuclear Medicine, School of Medicine and Health, Technical University Munich, Munich, Germany.; 3 German Cancer Consortium (DKTK), partner site Munich, a partnership between DKFZ and University Hospital rechts der Isar (MRI), Germany.; 4 Department of Radiation Oncology, School of Medicine and Health, Technical University Munich, Munich, Germany.; 5 Center for Translational Cancer Research (TranslaTUM), Technical University of Munich, Munich, Germany.; 6 Bavarian Cancer Research Center (BZKF), Munich, Germany.; 7 Ludwig-Maximilians-Universität München, Institute of Pathology, Munich, Germany.

**Keywords:** acute myeloid leukemia, CXCR4, allogeneic stem cell transplantation, endoradiotherapy, conditioning regimens

## Abstract

**Rationale:** Despite recent advances in the targeted therapy of AML, the disease continues to have a poor prognosis. Allogeneic hematopoietic stem cell transplantation (alloSCT) remains to be the curative therapy option for fit patients with high-risk disease. Especially patients with relapsed or refractory (r/r) AML continue to have poor outcomes. Myeloablative total body irradiation (TBI) based conditioning can be used in AML patients refractory to multiple lines of standard therapy, but the optimal conditioning regimen remains unclear for patients considered to be chemotherapy- refractory. Feasibility of C-X-C-motif chemokine receptor 4 (CXCR4)-directed endoradiotherapy (ERT) has previously been demonstrated in AML patients with CXCR4 expression on leukemic blasts.

**Methods:** Here, we report on a small cohort of seven AML patients refractory to multiple lines (range 3-7) of therapy, who received CXCR4-directed ERT with [^177^Lu]Pentixather in combination with TBI and chemotherapy prior to alloSCT. We report outcomes with a focus on toxicity, engraftment, the impact on the bone marrow (BM) niche and efficacy.

**Results:** In this intensively pre-treated group of patients, promising response (6 out of 7 patients) and engraftment (6 out of 7 patients) rates were observed. Histopathological analysis showed that niche compartments are spared and allow for engraftment to occur despite the combined ERT and TBI conditioning.

**Conclusion:** To the best of our knowledge, we report on the first seven patients who received CXCR4-directed ERT in sequential combination with TBI and chemotherapy, providing an effective, individualized conditioning regimen for intensively pre-treated r/r AML patients.

## Introduction

In recent years, therapeutic advances for acute myeloid leukemia (AML) were observed 1-4, but allogeneic hematopoietic stem cell transplantation (alloSCT) remains standard of care for fit patients with high-risk disease 5. However, relapsed or refractory (r/r) AML patients undergoing alloSCT have a poor prognosis, and relapse remains the main cause of AML treatment failure 6. Although a second alloSCT after relapse may achieve long-term remissions in selected AML patients, treatment options for patients with relapsed and uncontrolled AML are extremely limited 7. The increased risk of relapse after transplant is in part attributed to disease biology but also to incomplete eradication of malignant cells by induction, salvage and conditioning therapy 8, 9. Myeloablative conditioning (MAC) using total body irradiation (TBI) based protocols can be used in AML patients refractory to multiple lines of standard chemotherapy or in patients with AML relapse after first alloSCT who are considered chemotherapy-refractory. However, relapse rates are similar to those of other MAC regimens 10.

C-X-C-motif chemokine receptor 4 (CXCR4) is a G-protein coupled transmembrane receptor that regulates pivotal processes in hematopoiesis, immune response, embryogenesis and stem cell homing, together with its natural ligand CXCL12 11-14. Physiologically, CXCR4 is abundantly expressed in the hematopoietic system, especially in hematopoietic stem cells 15. In the context of malignancy, CXCR4 overexpression is commonly found in various types of cancer promoting proliferation and metastasis and mediating resistance to chemotherapy 16-20. The bone marrow (BM) niche plays an essential role in protecting healthy hematopoietic stem cells as well as leukemic blasts from cytotoxic stress. The CXCR4/CXCL12 axis is believed to mediate a protective microenvironment 17, 21. The protective niche is thought to be an important factor in disease relapse occurrence and mediation of drug resistance 22.

Radiolabeled peptides targeting CXCR4, such as [^68^Ga]Pentixafor and [^177^Lu]Pentixather were developed for diagnostic positron emission tomography (PET) and endoradiotherapy (ERT), respectively 23, 24. [^68^Ga]Pentixafor enables *in vivo* molecular CXCR4 imaging in a subset of AML patients 25. Pentixather is coupled with beta minus emitting radionuclides, like ^177^Lutetium or ^90^Yttrium which cause localized radiation damage within a few millimeters around the binding site. ERT of the CXCR4/CXCL12 axis is especially promising in refractory AML patients undergoing alloSCT, as it can destroy malignant cells directly but also dislocate them from their protective niche, rendering them more vulnerable to sequential or combined therapy approaches 26. The feasibility of CXCR4-directed ERT has previously been demonstrated in AML patients with CXCR4 expression on leukemic blasts 27. Naturally, hematological toxicity is a major concern of CXCR4-directed ERT, as it directly targets hematopoietic stem cells in the BM and the supporting niche by the “cross-fire effect”. In the context of alloSCT, the hematological toxicity itself is part of MAC and is bypassed by the stem-cell rescue, but damaging the hematopoietic niche may lead to engraftment failure or prolonged cytopenia 28.

## Methods

### Patient selection

Seven patients with r/r AML were treated at our center between February 2019 and October 2023. ERT with [^177^Lu]Pentixather was offered as compassionate use and in compliance with §37 of the Declaration of Helsinki. Written informed consent was obtained from all patients prior to treatment.

### [^68^Ga]Pentixafor PET/CT imaging

All patients underwent [^68^Ga]Pentixafor PET/computed tomography (CT) imaging to confirm *in vivo* CXCR4 expression on leukemic blasts. Increased tracer uptake of AML lesions as compared to normal tissue was visually determined by a board-certified nuclear medicine physician. Only patients with significantly visually increased tracer retention were considered eligible for ERT. Representative CXCR4 PET images of each patient are shown in Figure S1.

### Pre- and post-therapy dosimetry

Radiolabeling procedures of [^177^Lu]Pentixather are described in the supplemental material. All patients underwent pre-therapeutic dosimetry as follows: after intravenous injection of standard amounts of activity of 200 MBq (mean 198 ± 9.7 MBq, range 182-213 MBq) of [^177^Lu]Pentixather, whole-body scintigraphy or quantitative single photon emission (SPECT)/CT imaging was performed at 1 hour, 4 hours, 24 hours, 48 hours, and 7 days (d) post injection (p.i.). Post-therapeutic imaging was performed at the same time points following intravenous injection of the therapeutic activity of [^177^Lu]Pentixather. For whole-body scintigraphy, each scan was obtained at a speed of 12 cm/min on a dual-headed SYMBIA T6 (Siemens Medical Solutions, Erlangen, Germany) equipped with 9.5 mm NaI(Tl) crystals and medium-energy low-penetration collimators. A 12% and a 20% energy window were placed around the 208 keV and 113 keV photo-peaks of ^177^Lu, respectively. SPECT imaging was performed with 32 steps per head with 40 s acquisition duration per step using the 208 keV photo peak (12% width with upper and lower scatter windows with 15% and 10% width, respectively). A low dose CT was simultaneously acquired for attenuation and scatter correction. SPECT images were reconstructed in a 128x128 matrix using Flash 3D with 8 iterations and 4 subsets using CT-based attenuation and scatter correction. A camera- and isotope-specific calibration factor was applied to convert the images from counts to units of Bq.

For planar dosimetry, an extracorporeal background region of interest (ROI) was drawn for the liver and its content was scaled according to the size of the individual organ and subtracted. For the kidneys, the ROI was placed in the thigh. For the BM, the absorbed dose was determined by delineating the L2-L4 lumbar vertebrae following the guideline of the European Association of Nuclear Medicine (EANM) and the values were scaled accordingly 29, 30. The ROI for background subtraction was positioned adjacent to the BM. Following the administration of [^177^Lu]Pentixather and immediately before each planar whole-body scintigraphy, a probe counter measurement was conducted to normalize the counts to the residual activity in the patient's body at each time point. Count rates for each ROI were extracted using the open-source DICOM software OsiriX (version 5.1, 64-bit, Pixmeo, Geneva, Switzerland), and the geometric mean of the anterior and posterior counts for each ROI was calculated. The conversion of counts to activity was achieved through normalization using the probe measurements.

For dosimetry calculation with SPECT/CT, segmentations of volumes of interest (VOIs) were performed in MIM v7.2.8 software using their artificial intelligence (AI)-based segmentation model for CT images. Kidneys and liver were segmented using the AI-segmentation method and VOIs were copied to the co-registered SPECT images across imaging time points. For BM, three spherical VOIs were placed in the lumbar vertebrae L2 to L4 and total red BM was calculated above following the EANM guidelines 30.

Total organ activities were extracted and time-activity curves were fitted using either a mono- or bi-exponential fit function in MATLAB. Organ absorbed doses were calculated using phantom organ values from OLINDA/EXM v1.0 for whole-body scintigraphy and IDACDose 2.1 from the International Commission of Radiation Protection for SPECT/CT dosimetry using the organ time-integrated activities and patient-individual organ masses 31.

The results of the pre-therapeutic dosimetry were used to personalize the therapeutic amount of radioactivity of [^177^Lu]Pentixather based on scaling the absorbed dose per unit of administered activity (Gy/MBq) to organs at risk (maximum of 23 Gy to the kidneys and/or a maximum of 1 Gy to BM at the time of transplantation), yielding the amount of activity to be administered during therapy.

Post-therapeutic imaging was performed using the same protocols following intravenous injection of the therapeutic activity of [^177^Lu]Pentixather, using either whole-body scintigraphy or quantitative SPECT/CT imaging, followed by dosimetry calculations using the same approaches as described above.

### Treatment regime

**Figure 1:**
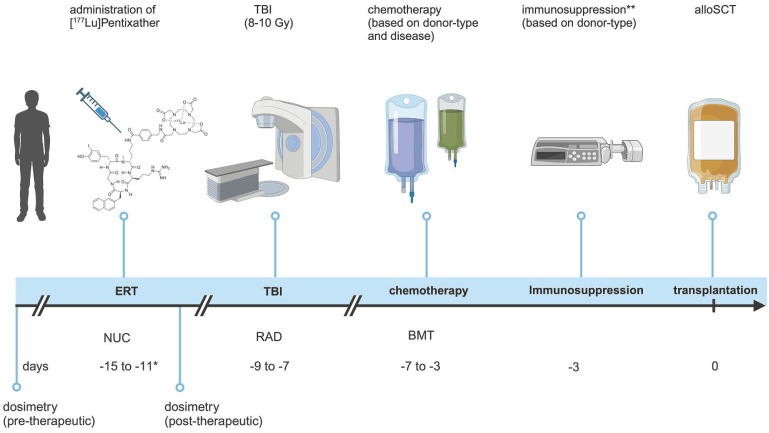
** Treatment schedule.** Schematic overview of the conditioning regimen. *Duration of in-patient stay varied based on German Radiation Protection Rules; **For patients with HID/MMUD, immunosuppression was started on day +3 instead. TBI = total body irradiation; alloSCT = allogeneic hematopoietic stem cell transplantation; NUC=nuclear medicine; RAD = radiation oncology; BMT=bone marrow transplantation. (Created in BioRender).

Conditioning prior to alloSCT consisted of [^177^Lu]Pentixather (mean activity of 12.4 ± 2.7 GBq, range 7.6-16.1 GBq) ERT on d-15 or d-14; TBI 8-10 Gy, depending on patient fitness and age, on d-9 to d-7 and chemotherapy, based on donor type (Figure 1).

ERT was performed in the department of nuclear medicine according to German Radiation Protection regulations. In addition, a 1000 mL saline solution containing 2% L-arginine and L-lysine was intravenously administered starting 30 minutes before ERT and continued for a total duration of 4 hours, as is commonly performed in patients with neuroendocrine tumors undergoing peptide receptor radionuclide therapy and has been adapted in prior studies with [^177^Lu]Pentixather 32, 33.

TBI was performed with twice-daily fractions of 2 Gy up to a desired dose of 8 (n=3) to 10 Gy (n=4). Patients were treated with a linear accelerator positioned supine and treated with lateral fields using 6-MV photons or with a tomotherapy system using intensity-modulated radiotherapy. The lungs were attenuated in all patients to a median dose of 8 Gy using the arms and brass compensators.

Chemotherapy regimens were as follows: fludarabine 30 mg/m^2^ d-5 to d-2 or cyclophosphamide 60mg/m^2^ d-5 and d-4 for matched unrelated donors (MUD) and matched related donors (MRD); fludarabine 30 mg/m^2^ d-6 to d-2 and cyclophosphamide 14.5 mg/m^2^ d-6 to d-5 for haploidentical donors (HID) or mismatched unrelated donors (MMUD). For patients with matched donors, immunosuppression consisted of rabbit antithymocyte globulin (5 mg/kg for MRD and 10 mg/kg for MUD) d-3 to d-1, mycophenolatmofetil and a calcineurin inhibitor. For HID and MMUD, post-transplant cyclophosphamide 50mg/kg on days 3 and 4, mycophenolatmofetil and a calcineurin inhibitor were used according to standard of care.

### Response assessment

AML response assessment was done by BM biopsy during routine follow-up according to standard of care as described in the European LeukemiaNet (ELN) recommendations 5. Patients with extramedullary disease were either staged by biopsy or by fluorodeoxyglucose (FDG)-PET-CT. Chimerism analysis was done by digital droplet polymerase chain reaction from the PB.

### Multispectral imaging

After antigen retrieval, immunofluorescence was performed using an Opal 6-Plex Detection Kit (Akoya Biosciences, Malborough, MA, USA) according to the manufacturer's protocol. The following antibodies were used MPO (Agilent DAKO, Santa Clara, CA, USA), CD34 (Agilent, DAKO), E-cadherin (Cell Marque, Darmstadt, Germany), CD42b (Agilent, DAKO), CD3 (Agilent, DAKO) and CD20 (Cell Marque). A PhenoImager (Akoya Bioscience) was used for multispectral imaging.

## Results

### Patient characteristics

Median patient age was 46 (range 42-57) years. Five patients had de novo AML and two had secondary (s)AML. Patients had previously received a median of four (3-7) lines of intensive therapy, including alloSCT in four patients (Table S1). Two patients were primary refractory and five patients relapsed after achieving a complete remission (CR). Prior to conditioning, one patient achieved morphologic leukemia-free state (MLFS) after salvage chemotherapy, all other patients were refractory and had active AML at the beginning of conditioning. Two patients had relapsed with extramedullary disease. Median hematopoietic cell Transplantation-Comorbidity Index (HCT-CI) Score was 1 (0-5) and at the start of conditioning, five patients had severe neutropenia with an absolute neutrophil count of <500/µl and four patients required broad-spectrum antibiotics at initiation of conditioning for uncontrolled infection. Median baseline creatinine was 0.8 mg/dl (0.4-1.1), and baseline bilirubin was 0.8 mg/dl (0.3-1.1). All patients received a peripheral blood (PB) stem cell graft with a median of 5.39 x 10^6^ (4.5-10.3) CD34+ cells/kg. Four patients received grafts from matched donors (3MUD and 1 MRD = 4 matched), two from HIDs and one from a MMUD (Table 1).

### CXCR4 PET imaging

**Figure 2:**
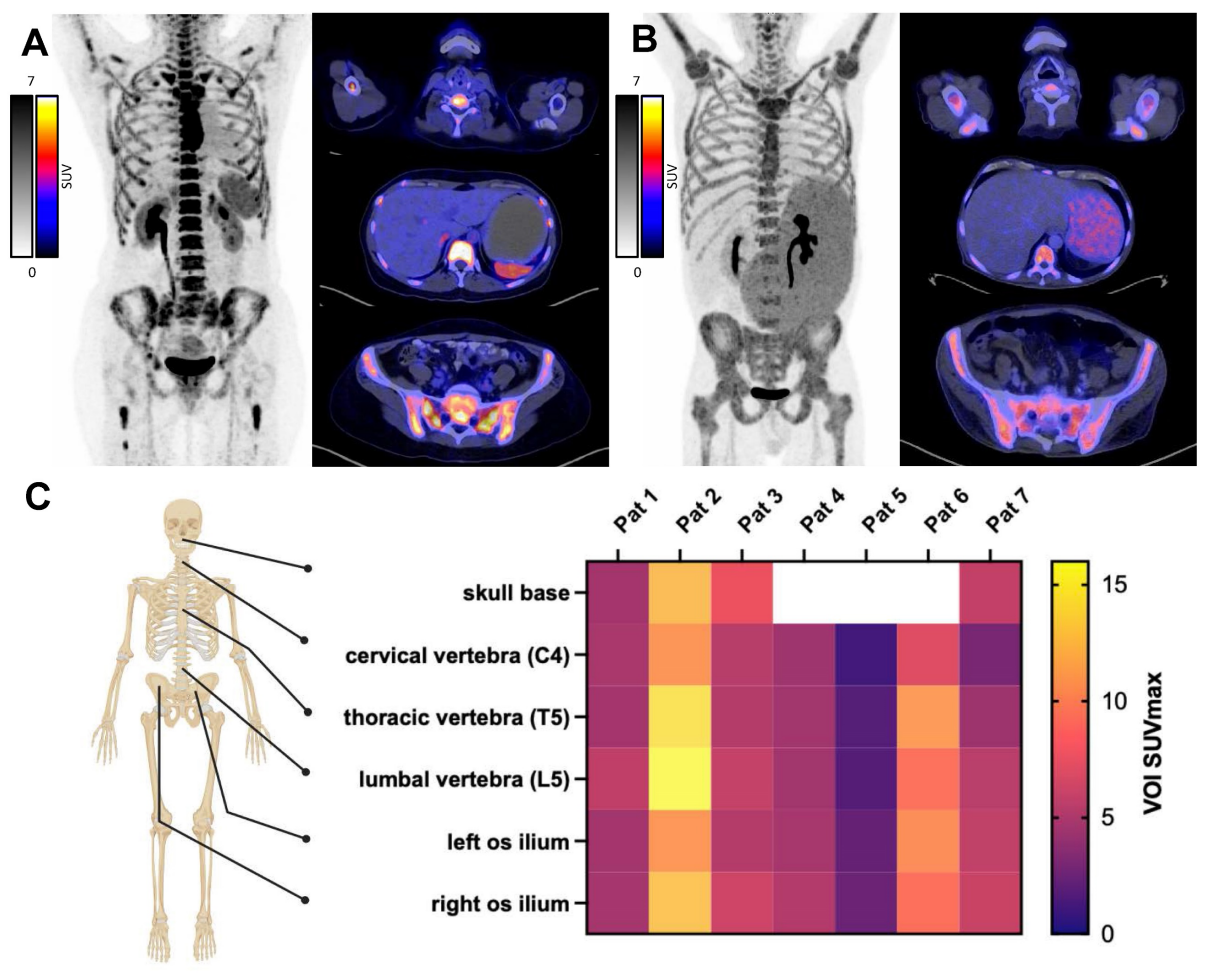
***In vivo* CXCR4 PET Imaging.** Pre-therapeutic PET/CT with the CXCR4 ligand [^68^Ga]Pentixafor to confirm *in vivo* CXCR4 expression. Patient examples with high (A) and moderate (B) CXCR4 expression on PET-imaging. C) SUVmax from pre-therapeutic CXCR4 imaging, measured at indicated sites. Patients 5 and 7 exhibited CXCR4 positive extramedullary disease. (Created in BioRender).

*In vivo* CXCR4 expression was confirmed by [^68^Ga]Pentixafor PET/CT in BM or extramedullary lesions (Figure 2) and patients with significantly visually increased tracer retention were considered eligible for ERT. Maximum standardized uptake value (SUVmax) was increased in skeletal system and was measured in the representative regions as follows: skull base 7.64 (range 4.61-12.47, not available for 3 patients), fourth cervical vertebra (C4) 5.19 (1.13-10.72), fifth thoracic vertebra (T5) 6.55 (1.78-14.42); fifth lumbar vertebra (L5) 6.93 (1.77-15.78) and left and right ilium 6.25 (2.31-10.74) and 6.67 (2.50-12.78), respectively. Of note, two patients (patient 5 and patient 7) had primary extramedullary disease, with a mean SUVmax of 1.90 (1.13-2.50) and 5.08 (2.93-6.09) across all measured sites.

### Dosimetry and conditioning

Radiolabeling details and methods for [^177^Lu]Pentixather can be found in Supplemental Material (Supplemental Methods and Table S2).

**Figure 3:**
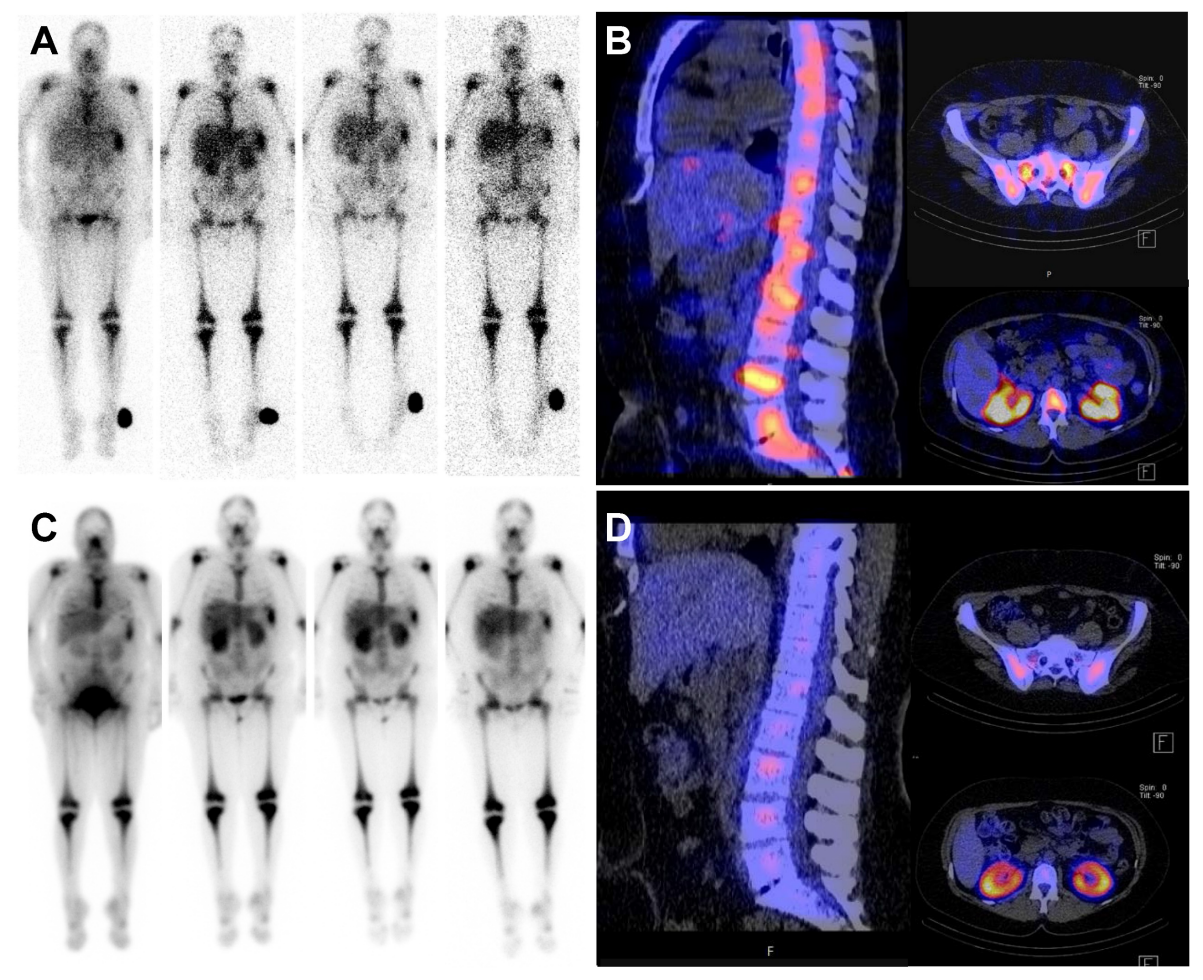
** Pre- and post-therapeutic dosimetry.** Pre- and post-therapeutic scintigraphy and SPECT/CT imaging of a 47 year old patient with refractory AML. Pre-therapeutic whole-body scintigraphy at 1 hour post injection (p.i.); 22h p.i.; 48h p.i. and 6 days p.i. (A, from left to right) for dosimetry including fused SPECT/CT (B). Post-therapeutic whole-body scintigraphy at 2 h, 24 h, 48 h and 6 d after administration of 14.4 Gbq [^177^Lu]Pentixather (C) and fused SPECT/CT (D).

Pre-ERT dosimetry was available for all patients. The median kidney absorbed dose across patients was 1.44 ± 0.70 Gy/GBq (range 0.73-2.97), the median liver absorbed dose was 0.40 ± 0.25 Gy/GBq (0.16-0.92), and the median BM absorbed dose was 0.42 ± 0.12 Gy/GBq (0.01-0.57). Post-therapy dosimetry was only available for 4 out of 7 patients. The median kidney absorbed dose across patients was 0.78 ± 0.22 Gy/GBq (range 0.68-1.21), the median liver absorbed dose was 0.34 ± 0.07 Gy/GBq (0.20-0.36), and the BM absorbed dose was 0.22 ± 0.12 Gy/GBq (0.10-0.41) (Table 2). Consequently, the potential therapeutic activity of [^177^Lu]Pentixather was limited either to a maximum of 23 Gy renal dose and/or a maximum of 1 Gy to BM at the time of transplantation (Figure 3). The mean injected therapeutic activity of [^177^Lu]Pentixather was 12.4 ± 2.7 GBq (range 7.6-16.1). No immediate reactions or adverse effects were observed during the obligatory 48-hour stay in the nuclear medicine ward post ERT as required by German Radiation Protection Rules. Patients were subsequently transferred to the SCT unit to receive TBI and chemotherapy.

### Toxicity

ERT, TBI and chemotherapy were generally well tolerated. No tumor lysis syndrome was observed during the conditioning period and no unexpected adverse events occurred. All patients developed fever and received broad-spectrum antibiotic and antifungal treatments during their hospitalization. Blood-stream infections were common and occurred in all seven patients and CT findings of pneumonia were seen in six patients. Mucositis II°-III° occurred in all patients. Four patients required intensive care unit (ICU) treatment due to infectious complications with hypotension or respiratory failure, two of these four patients required invasive ventilation during ICU stay. ICU admission occurred after a median 19 days (15-24) from the start of conditioning. An increase in serum creatinine of ≥ 1.5 folds was observed in three patients after conditioning therapy, in all three cases creatinine elevation was attributed to infectious complications. Among those patients, two required hemodialysis during their ICU stay. Hepatotoxicity was common, five patients developed I/II° and two patients III° hyperbilirubinaemia.

Three patients died during hospital stay after a median of 41 (30-83) days following conditioning. Among these patients, two died in the ICU due to septic shock (n=1) and acute respiratory distress syndrome (n=1) and one patient had refractory AML and died with palliative support. All deaths were considered to be either transplant-related or disease-related but not specific to ERT.

### Response and engraftment

One patient died prior to formal response assessment but achieved MLFS at day 15 after alloSCT. Five patients achieved complete remission with incomplete count recovery (CRi) and one patient was refractory and regenerated with 11% blasts in the PB at day eleven. Two patients with primarily extramedullary disease were either staged by FDG-PET-CT (for extramedullary disease) or by biopsy (for known chloroma of the skin). Both patients achieved complete remission. The recipient chimerism at or after day 28 was <1% (0.18% (0%-0.89%)) in all responding patients. Time to leukocyte recovery in the responding patients was 23 (12-28) days and platelet engraftment occurred at day 35 (12-55) in median, one patient had received a stem cell boost from the same donor after initial graft failure. Patients that were discharged had been hospitalized for a median of 41 days (35-89) for the transplantation period.

**Figure 4:**
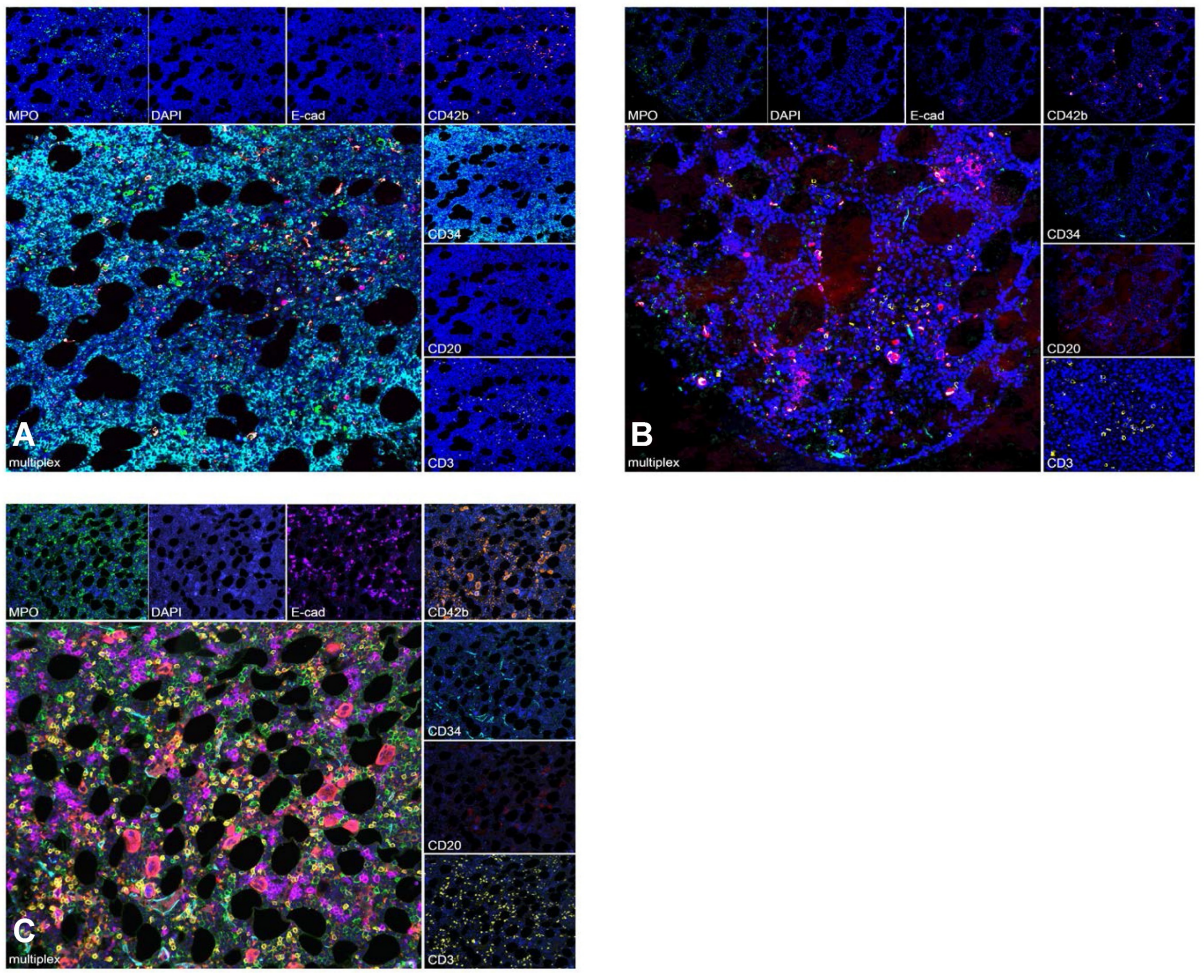
** Multispectral Imaging.** Multispectral image of an exemplary BM biopsy before treatment shows a compact infiltrate of CD34 positive AML blasts with reduced trilineage hematopoiesis (A). During aplasia, BM with clearance of AML blasts and markedly reduced trilineage hematopoiesis is detected (B). After engraftment, recovery of the BM microenvironment with normal distribution of hematopoiesis and the hematopoietic niche is shown (C). Granulopoesis is detected by MPO, erythropoiesis by E-cadherin and megakaryopoeisis by CD42b. CD20 highlights B- lymphocytes and CD3 T- lymphocytes. Myeloblasts are marked by CD34.

To further assess whether engraftment was impaired by the intensified conditioning regimen, multispectral imaging of exemplary BM sites biopsied before, during and after therapy was performed for patients with available samples. Before conditioning, a compact infiltrate of CD34-positive myeloblasts in a BM biopsy from a patient with AML with reduced trilineage hematopoiesis and few intermingled lymphocytes was seen. Following CXCR4-directed ERT, TBI and chemotherapy, hematopoiesis was markedly reduced and no residual blasts were detected. After engraftment, recovery of the BM microenvironment was observed with a normal distribution of hematopoiesis and the hematopoietic niche (Figure 4).

### Follow-up

Both patients with extramedullary only disease at the time of alloSCT had relapsed after three months. One patient had hematologic relapse, the other had isolated central nervous system (CNS) relapse. Both patients went to palliative care and died shortly after.

Two years after alloSCT, two patients were alive. One had developed CNS relapse twelve months after alloSCT but was successfully treated with donor lymphocyte infusions (DLI), 5-Azacitidine and radiotherapy. More than two years after alloSCT, the same patient developed pneumocystis jirovecii pneumonia and died in the ICU. One patient is in ongoing molecular CR more than four years after alloSCT. In the two patients with available follow-up, no long-term hepatic or renal toxicity was observed, as evidenced by normal creatinine and bilirubin levels in routine follow-ups (Table S3).

## Discussion

This retrospective analysis demonstrates that incorporating CXCR4-directed ERT with TBI and chemotherapy is feasible and results in promising response rates for patients with AML refractory to multiple lines of therapy.

TBI-based conditioning prior to alloSCT has proven highly effective in controlling AML, as evidenced by low relapse-associated mortality rates in various studies 34. However, this does not translate into improved OS, particularly for older r/r AML patients, due to substantial treatment-related mortality 35.

In AML, the CXCR4/CXCL12 axis is thought to play an important role in the BM-mediated resistance by providing a local protective environment. CXCR4-targeted ERT causes radiation-absorbed dose in AML-infiltrated organs, particularly the BM 36. Therefore, targeting CXCR4 in a sequential therapy approach and bypassing the hematologic toxicity by SCT is an intriguing concept for disease control in high-risk patients unable to achieve hematological remission before alloSCT. A significant concern with CXCR4-directed ERT is the potential destruction of the stem cell niche, caused by the so-called “cross-fire effect”, which could impair engraftment. Preclinical studies using a humanized murine leukemia model have demonstrated that CXCR4-directed ERT can effectively eliminate leukemic cells. Interestingly, the number of mesenchymal stromal cells (MSCs), the primary component of the stem cell niche, remained unchanged, although their proliferative capacity was impaired. Nonetheless, their overall functionality after ERT was not significantly affected, as post-alloSCT engraftment occurred within the expected timeframe 27. This may be partly explained by the low CXCR4 expression in MSCs and their resistance to radiation-induced cell death 37.

Retrospective analyses incorporating CXCR4-directed ERT in chemotherapy-based conditioning before auto- or alloSCT in hematologic malignancies such as T- and B-cell lymphomas, multiple myeloma and leukemia have confirmed adequate engraftment 33, 38-40. Our data reveal that combining CXCR4-directed ERT with TBI-based conditioning is also viable, with engraftment occurring within the expected range for all but one patient, who died prior to engraftment. Importantly, this retrospective analysis demonstrates for the first time in a patient setting that the mesenchymal compartment of the BM-niche retains its ability to support transplanted stem cells post-CXCR4-directed ERT.

Eradicating leukemic blasts is paramount in treating r/r AML patients to enable stem cell transplant engraftment and provide time for the immunogenic graft versus leukemia effect to develop. In this analysis, all but one patient achieved at least MLFS.

Other studies have demonstrated that lymphoid neoplasms exhibit higher CXCR4 expression compared to myeloid neoplasms such as AML 41. Integrating CXCR4-directed ERT into auto- or alloSCT strategies for heavily pretreated lymphoproliferative disorders, including lymphomas and multiple myeloma, has achieved remissions in all evaluated patients 33, 38-40. In myeloid neoplasms like AML, significant variability exists in the CXCR4 expression as determined by PET imaging, thus limiting the suitability of this approach to a specific subgroup 25. Notably, the two patients in this study who achieved long-term survival exhibited the highest CXCR4 expression within their AML manifestations. Consequently, *in vivo* CXCR4 expression, as assessed by PET imaging, may serve as a predictive marker for the therapeutic efficacy of CXCR4-directed ERT. However larger cohorts are needed to further support this observation.

In the context of AML comparable disease control and long-term survival rates have been reported in studies using chemotherapy-based conditioning regimens, such as clofarabine 42. Historical data show that patients with r/r AML have dismal prognosis with median OS of less than 12 months and for patients relapsing after alloSCT outcome is even worse 43, 44. However, most patients with AML who fail to respond to several lines of therapy either die before or are considered non-eligible for alloSCT, in consequence data on multi-refractory AML patients are scarce.

The observed toxicity profile highlights the aggressive nature of conditioning regimens for r/r AML, which, while potentially curative, pose considerable morbidity and mortality risks. Complications such as uncontrolled infection and organ damage rapidly accumulate in patients with uncontrolled disease who undergo multiple lines of treatment, often leading to a rapid decline in the patient's condition resulting in the inability to receive further intensive treatments. Given this context, some of the observed toxicities may be attributed to this difficult-to-treat cohort, in which all patients were heavily pre-treated and all but one patient had uncontrolled disease at the time of conditioning. Thus, the promising response rates observed in this cohort raise the question, whether applying this treatment approach in earlier lines, might yield better outcomes and lower toxicity rates when patients have undergone fewer previous lines of treatment. Consequently, a CXCR4-directed ERT approach in earlier therapy lines may not only enhance objective outcomes but also improve the quality of life for high-risk AML patients, as it counteracts the decline associated with multiple treatment lines and their long-term toxicities 45. However, long-term experience with other ERT approaches, such as PSMA- and SSTR-directed ERT, suggests that these therapies rarely can lead to hematological toxicities, including myelodysplastic syndromes, or organ dysfunctions such as renal insufficiency 46, 47. Although no such side effects have been observed in the studied cohort, the small number of patients and brief follow-up period preclude any definitive conclusions regarding long-term toxicity and efficacy following CXCR4-directed ERT.

Discussing other ERT strategies requires consideration of the pharmacokinetics and dynamics of the radiopharmaceuticals involved. ^188^Rhenium-labeled anti-CD66 and ^133^Iodine-labeled anti-CD45 have been successfully used in alloSCT conditioning for myeloid malignancies 48-50. The SIERRA phase III trial demonstrated superior response rates and survival with ^133^I-anti-CD45 compared to standard of care 51. A significant challenge is the timing between ERT and stem cell transplantation to ensure radiation decay and prevent harm to healthy stem cells. Conversely, shorter-lived ERT agents might be excreted faster and thus reduce toxicity but could compromise efficacy against deeply situated or protected leukemic cells. The use of Pentixather labelled with ^90^Y with its shorter half-life compared to ^177^Lu has been feasible and has significantly reduced the interval between ERT and SCT 33. However, ^90^Y has higher beta minus energies and a longer range that lead to increased irradiation of surrounding tissue. For example, ^177^Lu presents with a short beta minus maximum range of 2 mm, while ^90^Y presents with an 11 mm maximum range. Therefore, higher kidney damage could occur and has been observed in the treatment of neuroendocrine tumors with ^90^Y-DOTATATE compared to ^177^Lu-DOTATATE 52. The pharmacokinetics and potential benefits of other ERT strategies must therefore be carefully evaluated for the clinical setting of conditioning regimens to prevent additional toxicities.

## Conclusion

Data from this retrospective analysis indicate that CXCR4-directed ERT in sequential combination with TBI and chemotherapy offers an individually tailored treatment approach for patients with multi-refractory AML. No acute high-grade toxicities clearly attributed to ERT in addition to the conventional conditioning could be observed and stem cell engraftment was not impaired. Clearance of AML blasts was reached in the majority of patients (6 out of 7 patients). Future studies should refine CXCR4-directed ERT by dose optimization, patient selection and incorporation in earlier therapy lines for high-risk AML patients.

## Supplementary Material

Supplementary methods, figure and tables.

## Figures and Tables

**Table 1:** ** Patient Characteristics.** Table showing the baseline characteristics of the seven included patients. *Treatment lines were defined when a regimen change occurred due to relapse or progress of underlying disease. sAML = secondary acute myeloid leukemia; ELN = European LeukemiaNet; MUD = Matched unrelated donor; MRD = Matched related donor; MMUD = Mismatched unrelated donor; HID = Haploidentical Donor; MLFS = Morphologic leukemia-free state; RD = refractory disease.

Parameter	Value
Age, median (range)	46 (42-57)
Sex, n (%)	
male	4
female	3
AML type, n	
denovo	5
sAML	2
ELN 2022 risk group, n	
favorable	2
intermediate	3
adverse	2
Previous treatment lines*, median (range)	4 (3-7)
Previous alloSCT, n	4
Donor type, n	
MUD	3
MMUD	1
MRD	1
HID	2
Prior remission, n	
MLFS	1
RD	6
HCT-CI Score, median (range)	1 (0-5)

**Table 2:** ** Pre- and post-therapeutic dosimetry.** Estimated doses from pre- and post-therapeutic dosimetry delivered to indicated organs and the final injected activity. Post-therapeutic dosimetry was only available for 4/7 patients. Indicated doses are for ERT and should be considered as adjunctive to the sequential TBI. n.a.=not available, BM = bone marrow. *derived from SPECT-imaging

Patient	Kidney-Dose [Gy/GBq] (pre / post)	Liver-Dose [Gy/GBq] (pre / post)	BM-Dose [Gy/GBq] (pre / post)	Injected activity [GBq]
#1	1.59 / 0.87	0.29 / 0.20	0.42 / 0.14	12.0
#2	1.44 / 1.21	0.40 / 0.36	0.57 / 0.41	14.4
#3	1.73 / n.a.	0.47 / n.a.	0.47 / n.a.	13.0
#4	1.18 / 0.68	0.66 / 0.32	0.44 / 0.30	11.4
#5	0.73 / n.a.	0.16 / n.a.	0.11 / n.a.	12.2
#6	1.30 / 0.68	0.38 / 0.36	0.16 / 0.10	16.1
#7	2.97* / n.a.	0.92* / n.a.	0.01* / n.a.	7.6
