# Supplementary material for: CXCR4-directed endoradiotherapy with [177Lu]Pentixather added to total body irradiation for myeloablative conditioning in patients with relapsed/refractory acute myeloid leukemia
Source: Theranostics. 2025 Jan 1;15(1):19–29. doi: 10.7150/thno.101215 (PMC11667225; doi:10.7150/thno.101215)

Supplemental Material

CXCR4-directed endoradiotherapy with [^{177}Lu]Pentixather added to total body irradiation for myeloablative conditioning in patients with relapsed/refractory acute myeloid leukemia

Krischan Braitsch[1], Theo Lorenzini[2,3], Maike Hefter[1], Katrin Koch[1], Katharina Nickel[1], Jan C Peeken[4], Katharina S Götze[1], Wolfgang Weber[2,3,5,6], Anne Allmann[2], Calogero D'Alessandria[2], Julia Brosch-Lenz[2], Florian Bassermann[1,3,5,6], Martina Rudelius[7], Mareike Verbeek[1], Matthias Eiber[2,3,6,#] ,Peter Herhaus[1,#]

[1]Department of Internal Medicine III, School of Medicine and Health, Technical University of Munich, Munich, Germany.
[2]Department of Nuclear Medicine, School of Medicine and Health, Technical University Munich, Munich, Germany.
[3]German Cancer Consortium (DKTK), partner site Munich, a partnership between DKFZ and University Hospital rechts der Isar (MRI), Germany
[4]Department of Radiation Oncology, School of Medicine and Health, Technical University Munich, Munich, Germany.
[5]Center for Translational Cancer Research (TranslaTUM), Technical University of Munich, Munich, Germany.
[6]Bavarian Cancer Research Center (BZKF), Munich, Germany
[7]Ludwig-Maximilians-Universität München, Institute of Pathology, Munich, Germany

[#]equal contribution

Keywords: acute myeloid leukemia, CXCR4, allogeneic stem cell transplantation, endoradiotherapy, conditioning regimens

Supplemental Methods

Radiolabeling of [^{177}Lu]Pentixather

1000 µg Pentixather acetate were reconstituted in 1.0 mL TraceSELECT™ water (VWR International GmbH, Darmstadt, Germany). Of this solution, the required volume was added to ^{177}LuCl3 in 0.04 M HCl (EndolucineBeta®, ITM Radiopharm, Garching, Germany; activity concentration: 370 MBq/500 µl) based on the application. In particular, 20 ± 5 µg and 200 ± 50 µg of Pentixather were added to a solution of 1.0 GBq and 20.0 GBq of ^{177}LuCl3 for pre-therapeutic dosimetry or therapy application, respectively. [^{177}Lu]Pentixather was prepared using an iQS-TS synthesis module (ITM Radiopharm, Garching, Germany). Briefly, the necessary solution of Pentixather dissolved in 2.0 ml sodium ascorbate buffer (pH = 4-5), followed by automatic addition of the the solution of [^{177}Lu]Cl_3 in 0.04 M hydrochloric acid (ITM Radiopharm, Garching, Germany) to the reaction vial of aiQS-TS module cassette, and heated for 30 minutes at 95°C. The product was diluted with saline and passed through a sterile filter (0.22 µm, PALL Corporation, Merck) into a sterile vial. Radiochemical purity was determined using radio-TLC and analytical radio-HPLC. The administration of [^{177}Lu]Pentixather complied with The German Medicinal Products Act, AMG §13 2b, and the responsible regulatory body (Government of Oberbayern).

Radionuclide incorporation and radiochemical purity determination of [^{177}Lu]Pentixather

Radionuclide incorporation (RNI) [^{177}Lu]Pentixather was determined using radio-TLC and analytical radio-HPLC. Radio-TLC was carried out using Agilent iTLC silica gel impregnated chromatography paper (Agilent Technologies, Santa Clara, US) and 0.1 M sodium citrate at pH 5.0 as mobile phase. TLC-strips were analyzed using a Bioscan TLC analyzer (Eckert&Ziegler, Brussel, Belgium). Radiochemical purity (RCP) was further evaluated via radio high-performance liquid chromatography (HPLC) on a Prominence system with a Photo Diode Array detector (Shimadzu, Kyoto, Japan) and a GABI Star detector (Raytest, Straubenhardt, Germany). Eluents for all HPLC operations were water (solvent A) and acetonitrile (solvent B), both containing 0.1% trifluoroacetic acid. A Nucleosil C18 100-5 column (Macherey-Nagel, Germany) was used with a linear gradient of 15–90% B in 20 min, followed by 95% B for 5 min.

Analysis via radio-TLC revealed a RNI >99,5, and RCP as determined by radio-HPLC was 98,6 ± 1,0 with retention time of 12.07 min (Table S3). [^{177}Lu]Pentixather for this study was produced with a specific activity of 59,45 ± 19,51. The variability in specific activity was due to the performance of transfer of [^{177}Lu]Cl3 solution to the reaction vial.

Supplemental Tables

Table S1

Table showing previous treatment regimens, red arrows indicate relapse, purple arrows indicate refractory disease. Box size does not correlate with time spent on each treatment. Pat = patient; 7+3 = daunorubicin, cytarabine; HDAC = high dose cytarabine; ERT = endoradiotherapy; TBI = total body irradiation; alloSCT = allogeneic stem cell transplantation; HAM = high dose cytarabine, mitoxantrone; TAD = thioguanine, cytarabine, daunorubicin; AD = cytarabine, daunorubicin; AC = cytarabine, cyclophosphamide; AT = cytarabine, thioguanine ; DLI = Donor lymphocyte infusion; AZA = 5-Azacitidine; HU = hydroxyurea; Ven = venetoclax; GO = gemtuzumab-ozogamicin; CHOEP = cyclophosphamide, doxorubicin, vincristine, etoposide, prednisolone; FLAG-Ida = idarubicin, fludarabine, cytarabine; TEAM = bortezomib, cytarabine, gemtuzumab-ozogamicin; RTx = radiotherapy. *initial histology was t-cell lymphoma before extramedullary AML diagnosis was confirmed.

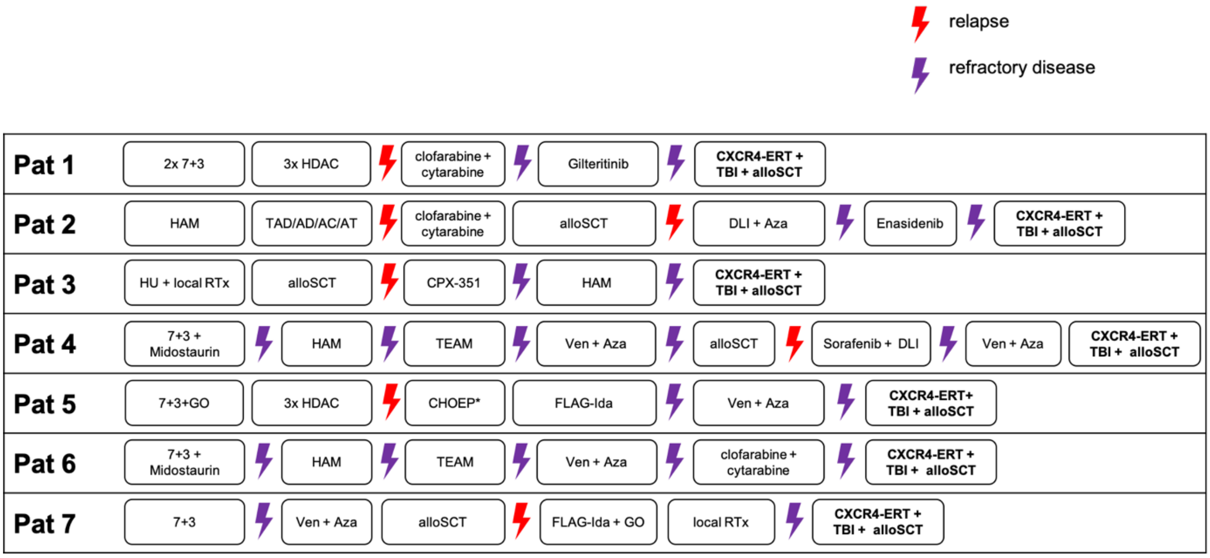

Table S2

Table reporting the results of radiolabeling of Pentixather with ^{177}Lu. The results of quality controls of [^{177}Lu]Pentixather are reported as % of radionuclide incorporation (RNI), radiochemical purity (RCP), radiolabeling yield (RLY). The specific activity is reported as MBq/μg of precursor used for the radiotracer production.

Patient	Injected activity [GBq]	Radiochemical purity (radio-HPLC, %)	Radionuclide incorporation (RNI, radio-TLC, %)	Radiolabelling yield (RLY, %)	Specific activity (MBq/μg)
1	12.0	n.a.	100	94	40.93
2	14.4	98.14	99.78	98.18	52.26
3	13.0	100	99.96	94	87.5
4	11.4	99.3	100	87.1	77.14
5	12.2	99.03	99.3	87.8	68.05
6	16.1	98.26	99.97	86.9	57.33
7	7.6	96.83	99.93	79.6	32.96

Table S3

Table displaying engraftment, response, toxicity and outcome for each patient. CTCAE = Common Terminology Criteria for Adverse Events; ICU = intensive care unit; alloSCT = allogeneic stem cell transplantation; OS = overall survival; nr = not reached; CR = complete remission; MLFS = morphologic leukemia-free state; RD = refractory disease; PCJ = *Pneumocystis jirovecii*

patient	leukocyte engraftment (days)	platelet engraftment (days)	remission after alloSCT	creatinine increased (CTCAE)	bilirubin increased (CTCAE)	ICU transfer	2 year OS	died during alloSCT	cause of death (at any time)	OS (days)
1	16	16	CR	0	I	no	yes	no	PJC pneumonia	784
2	26	55	CR	0	II	no	yes	no		>1500
3	nr	nr	MLFS	II	III	yes	deceased	yes	sepsis	41
4	12	12	RD	I	III	yes	deceased	yes	refractory disease	30
5	28	55	CR	I	II	yes	deceased	no	disease relapse	97
6	23	nr	CR	II	II	yes	deceased	yes	sepsis	83
7	12	14	CR	I	II	no	deceased	no	disease relapse	94

Supplemental Figure

Figure S1 – CXCR4 PET-CT images

CXCR4 PET maximum intensity projection (MIP) of all patients (numbers 1-7), shown as performed (unedited) in clinical routine.

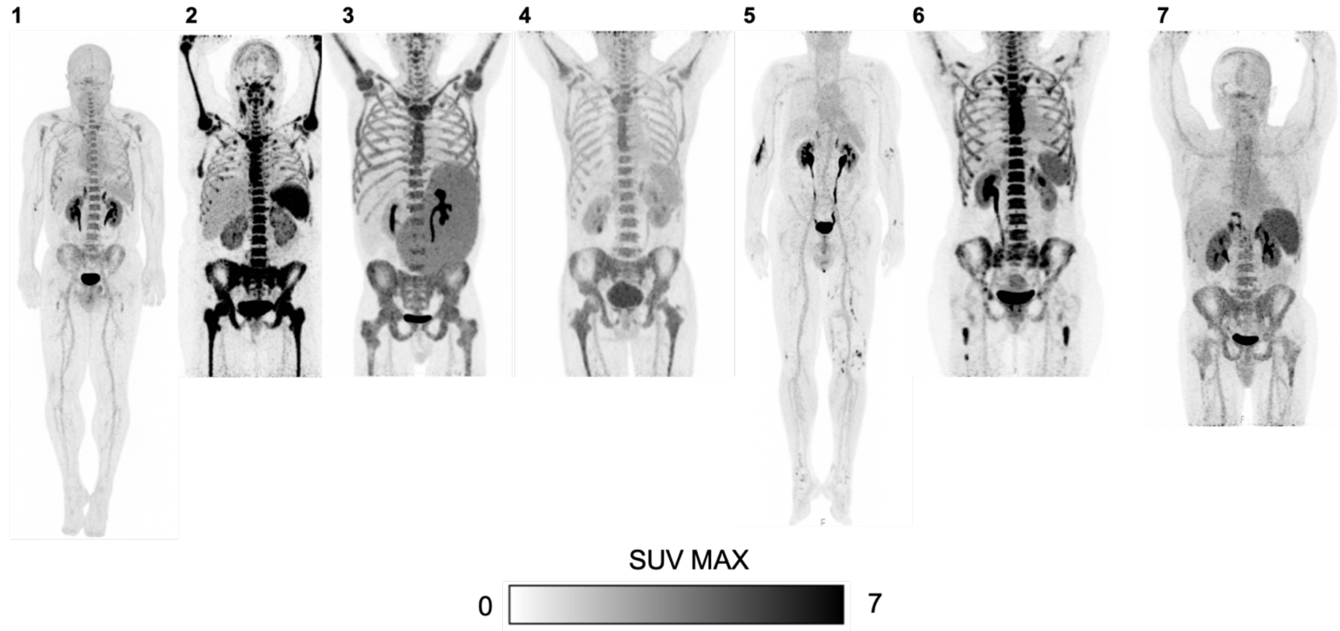

Supplement: Supplementary file 1 — Supplementary methods, figure and tables. [file thnov15p0019s1.pdf]
